# Juvenile depletion of microglia reduces orientation but not high spatial frequency selectivity in mouse V1

**DOI:** 10.1038/s41598-022-15503-0

**Published:** 2022-07-27

**Authors:** Dario X. Figueroa Velez, Miguel Arreola, Carey Y. L. Huh, Kim Green, Sunil P. Gandhi

**Affiliations:** 1 Department of Pathology, Children’s Hospital Boston, Boston, MA 02115 USA; 2 Department of Neurobiology and Behavior, University of California, Irvine, CA 92697 USA; 3 Center for the Neurobiology of Learning and Memory, University of California, Irvine, Irvine, CA 92697 USA

**Keywords:** Neuroscience, Striate cortex

## Abstract

Microglia contain multiple mechanisms that shape the synaptic landscape during postnatal development. Whether the synaptic changes mediated by microglia reflect the developmental refinement of neuronal responses in sensory cortices, however, remains poorly understood. In postnatal life, the development of increased orientation and spatial frequency selectivity of neuronal responses in primary visual cortex (V1) supports the emergence of high visual acuity. Here, we used the colony-stimulating factor 1 receptor (CSF1R) inhibitor PLX5622 to rapidly and durably deplete microglia in mice during the juvenile period in which increased orientation and spatial frequency selectivity emerge. Excitatory and inhibitory tuning properties were measured simultaneously using multi-photon calcium imaging in layer II/III of mouse V1. We found that microglia depletion generally increased evoked activity which, in turn, reduced orientation selectivity. Surprisingly, microglia were not required for the emergence of high spatial frequency tuned responses. In addition, microglia depletion did not perturb cortical binocularity, suggesting normal depth processing. Together, our finding that orientation and high spatial frequency selectivity in V1 are differentially supported by microglia reveal that microglia are required normal sensory processing, albeit selectively.

## Introduction

Across multiple sensory systems, experience guides the functional maturation of neuronal circuits during periods of heightened plasticity known as critical periods^1–5^. Abnormal experiences during these juvenile windows can perturb sensory coding and perception well into adulthood^6–15^. In vivo imaging studies of synaptic spines reveal that critical periods coincide with transient changes in spine stability^16–18^. Importantly, sensory deprivation during critical periods produces structural changes to synapses thought to reflect functional reorganization^16,17,19–21^. Thus, cell types that regulate synapse structure may play significant roles in the refinement of sensory circuit functional properties.

Microglia play multiple roles in shaping juvenile and adult brain circuits^21–23^. During development, microglia have been shown to modify synaptic connectivity through synapse elimination^24–29^, spine induction^30^, and trogocytosis^31^. In adults, microglia dampen cortical circuit activity through G_i_-dependent dynamics^32^ and by converting extracellular ATP into adenosine^33^, a potent depressant of neuronal activity^34^. The participation of microglia in synapse modifications and circuit activity suggests they may be involved in critical period mechanisms. In support, the elimination of microglia using inhibitors of the colony-stimulating factor 1 receptor (CSF1R)^35^ increases both synaptic connectivity and circuit excitability in primary visual cortex (V1)^36,37^.

During visual system development, the maturation of binocular visual processing has been thought to engage microglia mechanisms. In the early postnatal lateral geniculate nucleus (LGN), retinal activity drives the elimination of excessive binocular input from the retina leading to increased segregation of eye-specific visual pathways^38–41^. Synapse elimination by microglia has been implicated in the sculpting of retinal inputs to LGN^26–28^. While these studies provide strong evidence for the role of microglia in the anatomical refinement of visual circuits, they do not address function. Recently, it was found that disabling complement mechanisms prevents microglia-mediated pruning of developing retinal inputs to LGN^26^. Interestingly, this anatomical perturbation in LGN neither perturbs binocularity nor its plasticity downstream in V1^42^, highlighting the importance of assessing circuit function.

Beyond binocularity, the role of microglia in shaping functional properties of neuronal responses that support high acuity vision, such as orientation and spatial frequency selectivity^6,11,14,43–48^, has not been assessed. In the visual system, orientation-selective neurons exhibit strong preferences for the orientation of elongated visual stimuli^1,43,49–51^. In addition, neurons respond to a specific spatial frequency of repeating visual patterns^1,43,50–52^. Prior to eye-opening, V1 neurons have low orientation and spatial frequency selectivity. In the weeks following eye-opening, neurons increase their selectivity to stimulus orientation and their spatial frequency preferences shift toward finer stimuli^11,53–57^. The experience-dependent refinement of these tuning properties in V1 is strongly associated with the perceptual limits of visual acuity^14,45,47,58–60^.

To determine the contribution of microglia to the refinement and maintenance of visual responses, we carried out two-photon calcium imaging in V1 LII/III following long-term microglia depletion. We probed for multiple orientations and spatial frequencies through each eye to characterize neuronal selectivity^51,61^. While hyperexcitability was expected and observed, we were surprised to find that microglia depletion only reduced orientation selectivity. In contrast, we found that the developmental emergence of high spatial frequency tuned neurons occurred despite microglia depletion. Additionally, we find that the binocularity of V1 neurons is similar regardless of microglia survival. Together, these data provide compelling evidence that microglia are specifically required for the emergence of high orientation selectivity in V1.

## Results

### PLX5622 rapidly and sustainably eliminates microglia in juvenile V1

**Figure 1:**
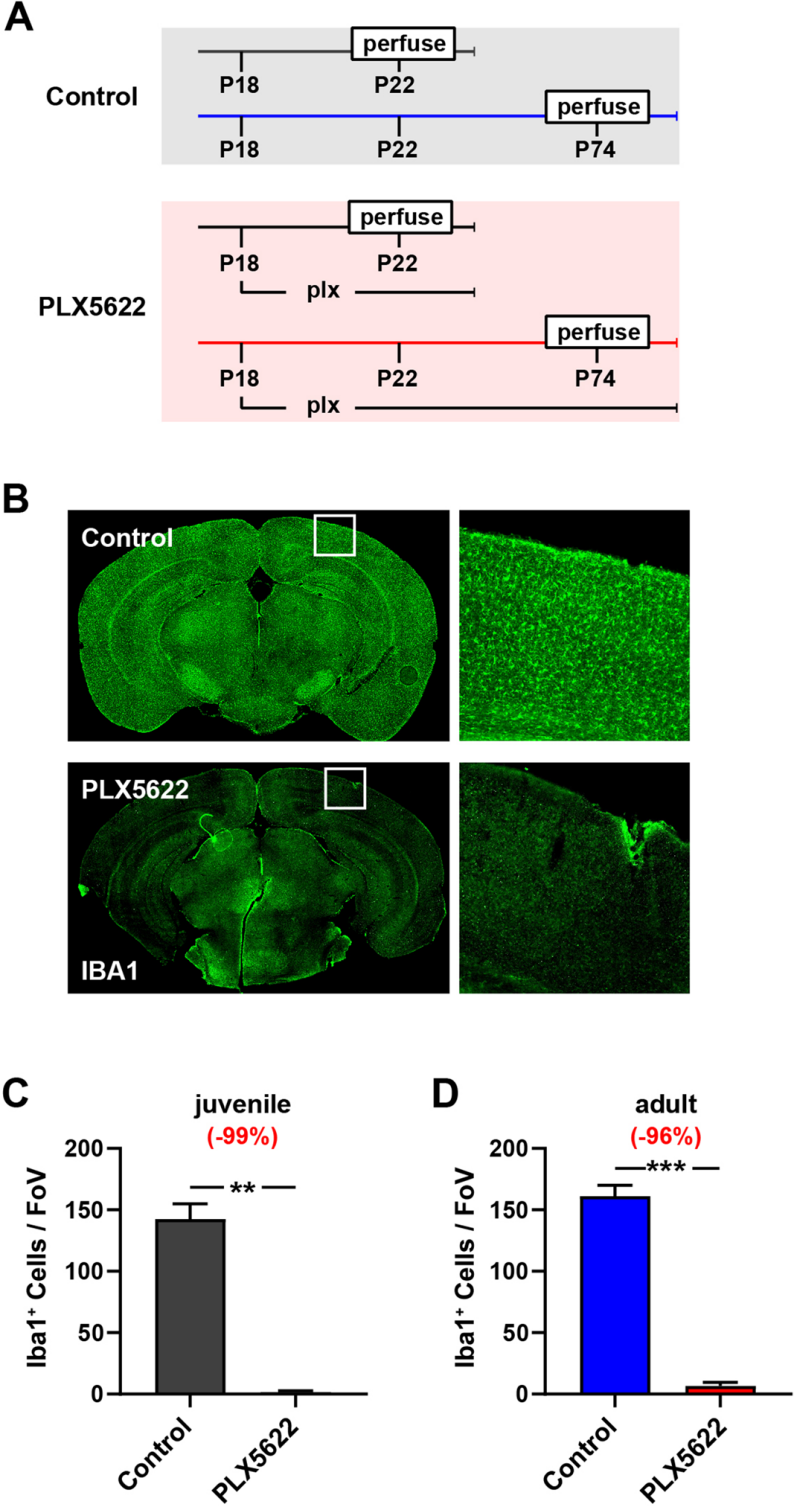
Rapid and sustained depletion of juvenile microglia with PLX5622 chow. (**A**) Timeline for assessing the extent of microglia depletion following PLX5622 chow administration. (**B**) Example images of P22 brain sections stained for Iba1 following control (top) and PLX5622 (bottom) chow diet. V1 is shown right. (**C**) The number of juvenile microglia is reduced by more than 99% in mice fed PLX5622 chow (Juvenile Control _(4 mice)_ = 142.5 ± 12.6 vs Juvenile PLX5622 _(4 mice)_ = 1.5 ± 1.2, Welsch’s t test: t = 11.2, df = 3.1, p = 0.0015). (**D**) Continued PLX5622 treatment maintains suppressed number of adult microglia (Adult Control = 161.2 ± 8.9 vs Adult PLX6722 = 6.8 ± 2.8; Welsch’s t test: t = 16.6, df = 6.0, p < 0.001). Error bars represent the S.E.M.

In adult mice, three days of treatment with the selective CSF1R inhibitor PLX5622 (1200 ppm in chow) eliminates most microglia from the adult brain^62^. We wanted to determine whether comparable depletion rates would occur in juveniles. To this end, we weaned P18 mice and fed them either control or PLX5622 chow diet until we collected brains for immunofluorescence staining (Fig. 1A). We visualized microglia using IBA1 staining (Fig. 1B) and found PLX5622 rapidly ablates > 99% of microglia from juvenile V1 (Fig. 1C). Importantly, continued PLX5622 diet sustained > 96% microglia depletion well into adulthood (Fig. 1D).

### Long-term microglia depletion increases evoked activity and reduces orientation selectivity in V1

**Figure 2:**
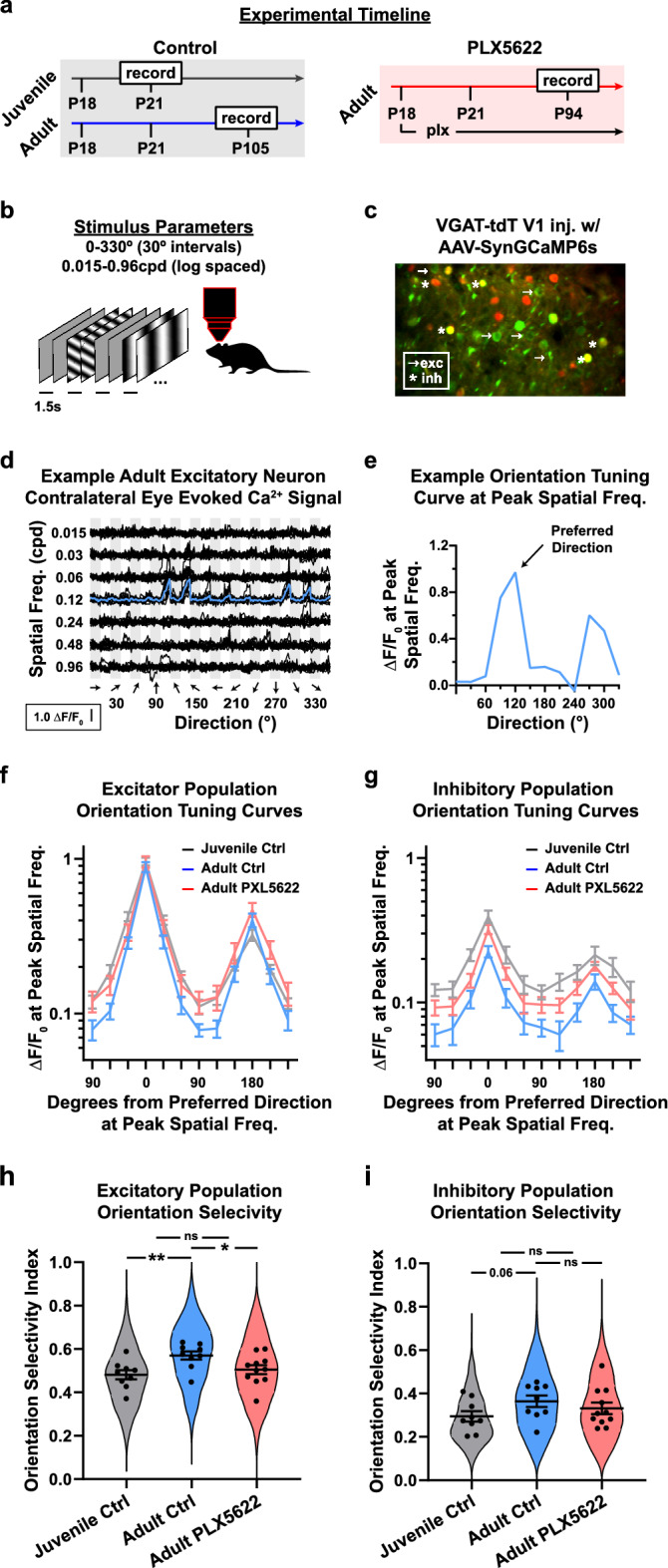
Reduced orientation selectivity following microglia depletion. (**a**) Timeline for two-photon imaging of tuning properties in normally reared juvenile and adult mice (top), and adult mice fed PLX5622 chow starting at P18 (bottom). (**b**) Experimental set up for assessing single-cell visually evoked activity using drifting gratings. (**c**) An example two-photon view of an AAV-Syn-GCaMP6s injected VGAT-tdT transgenic mouse V1. (**d**) Example visually evoked calcium signal to presentations of stimuli through the contralateral eye in adult V1. The x-axis is organized by grating direction. The y-axis is organized by increasing grating spatial frequency. Thin and thick black lines represent individual and trial averaged traces, respectively. The blue line represents the averaged responses to different directions at the neuron’s peak spatial frequency. This trial-averaged trace was used to generate the neuron’s orientation tuning curve and orientation selectivity. (**e**) The orientation tuning curve for the example neuron in (**d**). (**f**,**g**) Individual tuning curves were shifted around the preferred direction and averaged for each animal. (**f**) The population orientation tuning curves for excitatory neurons in juvenile (grey), adult (blue), and adults on PLX5622 chow (red). (**g**) The population orientation tuning curves for inhibitory neurons in juvenile (grey), adult (blue), and adults fed PLX5622 chow (red). The OSI for excitatory (**h**) and inhibitory (**i**) neurons. Violin plots represent the population distribution in juvenile (grey) and adult control (blue) mice, and adults on PLX5622 (red). Black circles represent an animal’s mean OSI. (**h**) During normal development, OSI increases in excitatory neurons (Juvenile Control = 0.48 ± 0.02 vs Adult Control = 0.57 ± 0.02, p = 0.007). The OSI of adult PLX5622 (0.50 ± 0.02) was lower than adult controls (p = 0.024) and comparable to juvenile control (p = 0.525). (**i**) During normal development, there is an increase in the OSI for inhibitory neurons (Juvenile Control = 0.29 ± 0.02 vs Adult Control = 0.36 ± 0.03, p = 0.05). The OSI of adult PLX5622 (0.33 ± 0.03) mice was in between but not different from control juveniles (p = 0.294) and adults (p = 0.330). n_JuvenileControl_ = 9 mice, n_AdultControl_ = 9 mice, n_AdultPLX5622_ = 11 mice. Error bars represent the S.E.M.

The improvement in visual acuity after eye-opening coincides with the maturation of V1 tuning properties^11,47,59,63,64^. To determine the role of microglia in the experience-dependent refinement of V1 tuning, we used two-photon microscopy to record visually evoked calcium signals in control juvenile and adult mice, and in adult mice fed PLX5622 (Fig. 2a). At the time of recording, mice were presented with a battery of visual stimuli consisting of drifting gratings spanning 7 spatial frequencies (0.015–0.96 cpd spaced logarithmically) and 12 directions (0°–330°) (Fig. 2b). Stimuli were randomized and presented 10 times to each eye separately. Inhibitory neurons were identified based on expression of the red fluorescence protein tdTomato in cells that express the vesicular inhibitory amino acid transporter (VGAT) (Fig. 2c).

The typical neuron in adult V1 is strongly responsive to few drifting gratings that are opposite in directions^43,50,65^ (Fig. 2d). The orientation tuning curve for the example neuron was calculated by taking the responses for all orientations at the spatial frequency which evokes the strongest response (Fig. 2e). To visualize orientation selectivity across the population of excitatory (Fig. 2f) and inhibitory (Fig. 2g) neurons, orientation tuning curves were shifted and centered around the preferred direction. The average of these population tuning curves reveals that the levels of evoked activity across most directions was higher in juveniles (grey) and in adults fed PLX5622 (red) than in control adults (blue) (Fig. 2f,g). To quantify a neuronal orientation selectivity, we calculated their orientation selectivity index (OSI) which provides a summary value that considers the responses at all orientations. A neuron with an OSI of 0 responds to all orientations with equal magnitude. A neuron with an OSI of 1 responds to only one orientation. In agreement with previous studies, excitatory neuron OSI increases between juvenile and adult ages (Fig. 2h). The OSI of adult mice fed PLX5622 chow were comparable to those of juvenile and not adult mice (Fig. 2h). Similarly, the OSI of inhibitory neurons of mice fed PLX5622 chow was not different from juveniles (Fig. 2i).

### Microglia are not required for high spatial frequency selectivity and maintenance of normal binocularity

**Figure 3:**
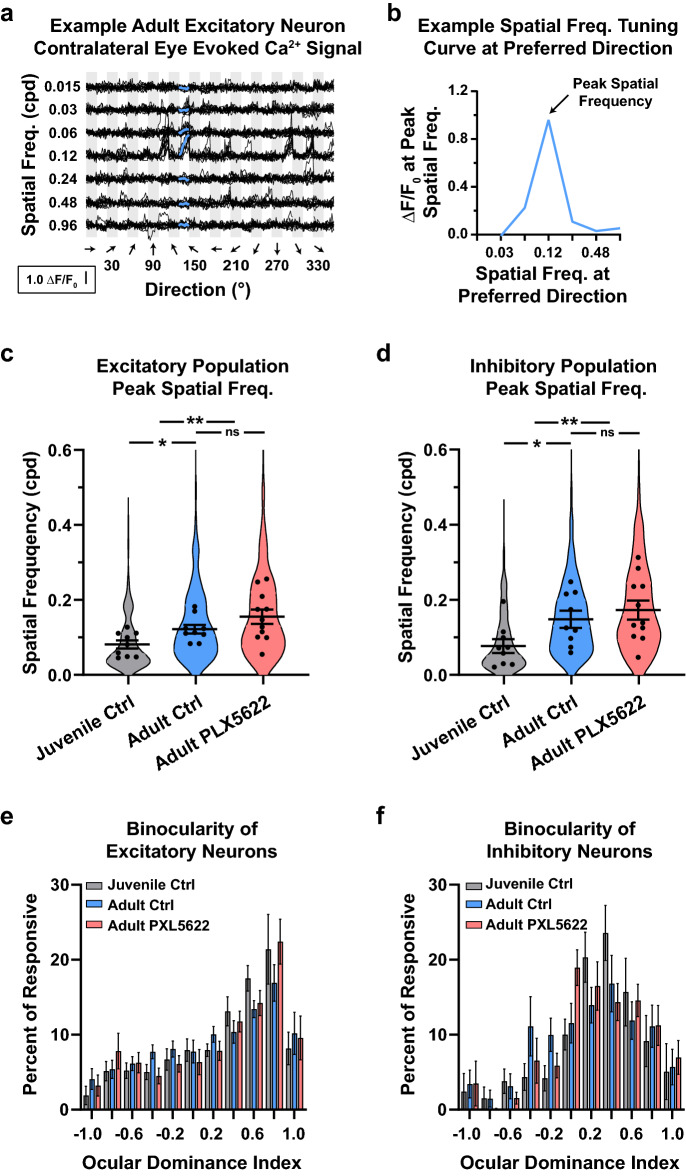
Microglia are not required for the developmental emergence of high spatial frequency tuning nor maintenance of normal binocularity in V1. (**a**) Example visually evoked calcium signal to presentations of stimuli through the contralateral eye in adult V1. The x-axis is organized by grating direction. The y-axis is organized by increasing grating spatial frequency. Thin and thick black lines represent individual and trial averaged traces, respectively. The blue line at 120° represents the averaged responses to different spatial frequencies directions at the neuron’s preferred direction. This trial-averaged trace was used to generate this neuron’s spatial frequency tuning curve and peak spatial frequency. (**b**) The spatial frequency tuning curve for the example neuron in (**a**). The peak spatial frequency for excitatory (**c**) and inhibitory (**d**) neurons. Violin plots represent the population distribution in juvenile (grey) and adult control (blue) mice, and adults on PLX5622 chow (red). Black circles represent an animal’s mean peak spatial frequency. (**c**) During normal development, excitatory neurons shift toward higher spatial frequencies (Juvenile Control = 0.08 ± 0.01 vs Adult Control = 0.12 ± 0.01, p = 0.035). The peak spatial frequency of mice fed PLX5622 (0.16 ± 0.02) was higher than juvenile (p = 0.002) and comparable to adult control mice (p = 0.253). (**d**) Like their excitatory counterpart, inhibitory neurons shift toward higher spatial frequencies during normal development (Juvenile Control = 0.08 ± 0.02 vs Adult Control = 0.15 ± 0.02, p = 0.043). The peak spatial frequency of mice fed PLX5622 (0.18 ± 0.03) was higher than juvenile (p = 0.006) and comparable to adult control mice (p = 0.450). Histogram of ocular dominance index for excitatory (**e**) and inhibitory (**f**) neurons in juveniles (grey), adults (blue), and mice lacking microglia (red). Microglia depletion did not alter the established binocularity of neurons in V1 (Juvenile Control = 0.45 ± 0.08 vs Adult Control = 0.30 ± 0.08 cpd, vs Adult PLX5622 = 0.36 ± 0.11). n_JuvenileControl_ = 9 mice, n_AdultControl_ = 9 mice, n_AdultPLX5622_ = 11 mice. Error bars represent the S.E.M.

The emergence of high spatial frequency tuned neurons in adult visual circuits is a hallmark of critical period development. The typical neuron in adult V1 is strongly selective for stimulus that is mid-to-high spatial frequency (Fig. 3a)^51,64^. The spatial frequency tuning curve for the example neuron was calculated by taking the responses for all spatial frequencies at the preferred direction (Fig. 3b). The spatial frequency with the highest response magnitude is considered that neuron’s peak spatial frequency. During normal development, there is shift toward higher peak spatial frequencies in both excitatory (Fig. 3c) and inhibitory (Fig. 3d) neurons. Microglia depletion did not prevent the developmental shift toward higher spatial frequencies in both neuronal populations (Fig. 3c,d).

Another hallmark feature of critical period development is the emergence and functional stability of binocularity. Binocular neurons generally respond more strongly to stimulation shown through one eye versus another. This characteristic can be quantified using the ocular dominance index (ODI). A value of − 1 and 1 ODI represents a cell that responds only to ipsilateral and contralateral eye stimulation, respectively. Across all groups, excitatory neuron evoked activity (Fig. 3e) is less binocular (ODI biased toward 1) than their inhibitory counterpart (Fig. 3f). The conserved pattern of stronger binocularity in inhibitory neurons suggests microglia depletion does not perturb binocular processing in V1.

## Discussion

The reorganization of synaptic connectivity is thought to underlie the experience-dependent maturation of sensory circuits^21^. In the visual system, while microglia support postnatal connectivity through synapse elimination^26–29^, their contribution to the development of neuronal tuning is unclear. In this study we addressed what role microglia play in the refinement of neuronal tuning during a critical period for V1 development by depleting them using the CSF1R inhibitor PLX5622. We find that microglia elimination elevated evoked activity reduced orientation selectivity while sparing high spatial frequency selectivity and binocularity. Our observations provide a dissociation for the mechanisms that support orientation selectivity from those that support high spatial frequency selectivity.

We chose to use the CSF1R inhibitor PLX5622 because of its well-documented efficacy in rapid microglia ablation^66–69^. Due to the pharmacological nature of the paradigm, other CSF1R expressing cells in the body will have altered CSF1R signaling, however, microglia and brain macrophages appear uniquely dependent on CSF1R signaling for survival^70^, and thus gross elimination of other myeloid populations does not occur. Genetic approaches for the elimination of microglia have also been developed, and several Cre driver lines developed that can selectively target microglia over other myeloid populations, such as TMEM119^71^ and Hexb^72^. Combination of these lines with Cre-dependent diphtheria toxin expression mice rapidly kills the microglial population, but results in a cytokine storm and rapid repopulation from surviving cells^73–75^. Neither a cytokine storm, nor repopulation occur with CSF1R inhibitor treatments that deplete microglia^70^. It should be noted that while CSF1R inhibitors represent the current best approach for microglial depletion, they result in brain-wide elimination of microglia^35^. Thus, we cannot conclude that our observations in V1 are due to the local loss of microglia. Moreover, our study cannot exclude the role of other myeloid cells in supporting high V1 orientation selectivity. The combination of a Cre-dependent, secreted CSF1R inhibitor protein^76^ with local delivery of an AAV-Cre under neuron-specific promoters^77^ and serotypes^78^ may prove useful to test whether sustained, local depletion of microglia in V1 reproduce our results.

Our data suggest a selective and necessary role for microglia in increasing orientation selectivity. One explanation is that microglia function like inhibitory neurons, which shape cortical tuning by increasing the threshold for excitability, thus making excitatory spiking output more selective^56,79^. Therefore, in the absence of microglia, neurons may become more excitable and effectively increase responsiveness to non-preferred stimuli, which could reduce orientation selectivity. Indeed, in a mouse model for Angelman syndrome, increased intrinsic neuronal excitability coincides with a reduction of orientation selectivity in V1^80^. In support of an excitability mechanism, the spontaneous synaptic transmission of excitatory and Parvalbumin (PV) inhibitory neurons in V1 has been shown to increase following microglia depletion^36,37^. Thus, the microglia mechanisms that regulate circuit-wide excitability may contribute to the developmental refinement of tuning properties.

Orientation and spatial frequency selectivity are largely separable over time and space^81–83^. In addition, some synaptic molecules have been recently found to be required for the refinement of select cortical response properties^59,84–86^. It is therefore possible that microglia recognize and modify synapses with specific tuning properties. Indeed, in the developing retinogeniculate system, activity-associated molecular markers on neurons tell microglia which synapses to eliminate^24,26,27^. Thus, our finding that microglia are selectively required for the refinement of orientation selectivity may reflect the ability of microglia to recognize unique synaptic tags linked to tuning properties.

In contrast to neurons, microglia are smaller and tile the brain with little overlap of their motile processes. Therefore, the density of the tiling may set the spatial scale at which microglia can engage in differential synapse elimination. It has been established that, at least for orientation selectivity, the preferences of synaptic inputs to an individual neuron only weakly predict its overall orientation preference^87,88^. Recently, it has been found that synapses with orientation preferences like those of the parent neuron are tightly correlated in time and cluster together along the dendritic arbor^89–91^. The clustering of functionally similar synapses creates hotspots of activity that may therefore differentially engage microglia-mediated synapse elimination and induction. If the binocularity and spatial frequency selectivity of synapses along the dendritic arbor are more homogenously distributed, microglia may be engaged in a manner which does not bias the overall distribution of those tuning properties. To better understand the role of microglia in sensory circuit development, future studies should probe for a relationship between microglial contact and the distribution of synaptic tuning properties across the dendritic arbor.

To our knowledge, this study is the first to measure the developmental refinement of tuning in V1 layer II/III GABAergic neuron populations. In agreement with others, we find that adult inhibitory neurons are less selective to stimulus orientation than excitatory neurons^50,92,93^. Our analysis did not separate by inhibitory type. Previous developmental studies show Parvalbumin inhibitory (PV) neuron orientation selectivity reduces during juvenile development^56,94,95^. Here, we show that the inhibitory population becomes more selective to stimulus orientation. This may reflect a divergence in the functional development of PV neurons from the other major class of inhibitory neurons, Somatostatin-expressing (SST) cells^96–98^. Indeed, SST cells in adult V1 display orientation selectivity that is comparable to excitatory neurons^99^. If immature SST neurons have reduced orientation selectivity, our observed developmental increase of orientation selectivity for inhibitory neurons in layer II/III may be driven by SST development. Future studies may reveal more specific effects of microglia depletion on different subtypes of cortical cells.

While our observations were limited to V1, the effects of microglia depletion on the maturation of orientation selectivity may extend to other visual circuits. Although high orientation selectivity predominately emerges in V1 of the primary visual pathway, superior colliculus (SC) neurons of the extrageniculate visual pathway exhibit weak selectivity to stimulus orientation^100^. The SC is a visual circuit crucial for spatially guided oriented responses^101,102^. Importantly, distinct SC cell types^103^ support predator-avoidance^104^ and prey-capture behavior^105^. If microglia support orientation selectivity across visual circuits, their depletion may perturb orientation selectivity in SC which could disrupt prey-predator behavior.

To date, the relationship between microglia synapse elimination and neural circuit function has been limited to broad changes in circuit activity. Importantly, until this study, the role of microglia in the development of neuronal tuning during normal visual experience had not been directly assessed. Here we find a selective but lasting impact on circuit function when microglia are eliminated starting in juvenile development. Although high spatial frequency tuned neurons emerge in microglia depleted V1, blunted orientation selectivity alone likely impairs the animal’s visual acuity. Our study provides compelling evidence for a key role for microglia in the postnatal development of sensory systems and suggests that microglia deficiencies during postnatal critical periods may lead to subtle but long-lasting deficits in sensory processing.

## Experimental methods

### Animals

To visualize inhibitory neurons, mice harboring the Cre-dependent tdTomato reporter (Ai14, JAX 007914) were crossed with mice expressing Cre recombinase under the control of the Vesicular GABA Transporter (VGAT) promoter (VGAT-ires-Cre^106^, JAX 028862). Mice were kept on a light/dark cycle (12/12 h). All experiments took place during the light cycle and used both male and female mice. All experimental protocols and procedures were approved by and followed the guidelines of the Institutional Animal Care and Use Committee (IACUC) at the University of California, Irvine. This study is reported in accordance with ARRIVE guidelines.

### PLX5622 administration

PLX5622 was provided by Plexxikon Inc. and formulated in AIN-76A standard chow by Research Diets Inc. at 1200 mg/kg. All mice were weaned at P18, and littermates split into two groups: (1) PLX5622 chow, and (2) control chow. To encourage feeding, 1 pellet was dopped per mouse per day until the fourth postnatal week. This was particularly important for recently weaned mice which have difficulty reaching the food hopper.

### Surgical preparation

Mice were anesthetized with Isoflurane (Patterson Veterinary) in O_2_ (2% for induction, 1–1.5% for maintenance). Injectable Carprofen (10 mg/kg s.c., Zoetis) was administered to provide perioperative analgesia. Ringer’s lactate solution (0.5 mL/20 g/h, s.c.) was given to replace fluid loss. Eyes were protected from dehydration with sterile eye ointment (Rugby, Livonia, MI). All surgical tools were sterilized using a hot glass bead sterilizer (Germinator 500). Mice recovered in their home cage over a warm heating pad until normal eating and grooming behavior resumed. Post-operative care consisted of daily Carprofen injections for at least 2 days following headplate implantation and 5 days following cranial window surgeries.

#### Headplate implantation

Following hair removal and sterilization with Povidone-iodine, the scalp was covered in topical 2% lidocaine hydrochloride jelly (Akorn) for at least 5 min. The scalp and underlying connective tissue were removed to expose the parietal and interparietal bone. Lidocaine hydrochloride (1%) was injected into the right temporalis which was subsequently detached (posterior half) from the skull. The skull was dried using ethanol (70% in DI water) and a thin layer of the tissue adhesive Vetbond (3 M) was applied. Custom-printed titanium headplates (Star Rapid) were attached to the skull using the acrylic resin Ortho-Jet BCA (Lang Dental).

#### GCaMP6s virus delivery

To monitor neuronal activity, we used the genetically encoded calcium indicator GCaMP6s (Penn Vector Core) under the neuron specific promoter Synapsin^77^ (1 × 10^12^ GC/mL). For juvenile recordings, neonates (P2-5) were ice-anesthetized and a beveled glass micropipette (~ 100 µm tip diameter) lowered perpendicular to ~ 3.5 mm lateral and 1 mm anterior the confluence of sinuses. The micropipette was advanced quickly into cortex to a depth of ~ 700 µm below the surface and a hydraulic manipulator (Narishige, MO-10) used to inject the virus (200–400 nL, 200 nL/min). Subsequent retinotopic maps (see “ntrinsic signal optical imaging” section) were used to confirm virus was injected into binocular V1. For adult recordings, viral delivery to V1 was guided by retinotopic maps within 1 week following the headplate implantation. A burr hole was made over central bV1 until a small region of dura was exposed. A syringe tip was used to nick the dura to prevent the rupturing of blood vessels and tissue damage from the micropipette pushing down on the brain prior to penetration. Virus was injected at a depth of 350 µm below the dura at 6.66 nL/min. Mice with any brain vessel ruptures were not used for this study. We found that multiple thin layers of Vetbond (immediately wicked away with Kimwipes) over the burr hole protected the skull and did not cause visible damage as noted after skull removal during the cranial window surgery which took place 3–4 weeks later.

#### Cranial window implantation

In addition to Carprofen, mice received a single injection of Dexamethasone (5 mg/kg, s.c.) to prevent brain edema. A small craniotomy (3 mm) was centered over V1. An additional 1 mm outer skull region was thinned so that a 4 mm glass coverslip (World Precision Instruments, Sarasota, FL) would sit over the exposed brain and pressed against thinned bone surrounding the craniotomy. GELFOAM sterile sponges (Patterson Veterinary) pre-soaked in sterile saline were used to absorb bleeding from the dura following skull removal. The glass was held down manually using forceps and the edges affixed to the skull first with a thin layer of Vetbond and then with acrylic resin. Mice with operative or post-operative bleeding in the parenchyma were not used. Some mice had had minor post-operative dural bleeding. Dural bleeding that did not completely clear within three days normally led to bone growth and poor imaging clarity. Therefore, mice were euthanized if their post-operative bleeding did not clear within three days.

### Imaging procedures

All recordings were done in awake, head-fixed mice on a smooth tablet surface. All stimuli were presented on a gamma-corrected 24-inch LED monitor (Asus VG248, 60 Hz refresh rate, 20 cd/m^2^), placed 25 cm from the mouse's eyes.

#### Intrinsic signal optical imaging

Hemodynamic responses were imaged using a SciMedia THT macroscope (Leica PlanApo 1.0 × , 6.5 × 6.5 mm imaging area) equipped with an Andor Zyla sCMOS (30fps). An LED driver (DC4104 Thorlabs) was used for illumination. First, a reference image of the surface vasculature was captured using green light (530 nm). Next, the camera was focused ~ 600 μm beneath the skull surface. A red filter was inserted into the path and a red light (617 nm) used to evenly illuminate V1. The stimulus presented in all experiments spanned the central 30° of the mouse’s visual field and consisted of a contrast modulated sweeping noise stimulus periodically every 20 s. Each recording trial consisted of 10 presentations at 0° and 180°. The stimulus was generated by multiplying a band-limited (< 0.15 cpd, < 4 Hz) binarized spatiotemporal noise movie with a one-dimensional Gaussian spatial mask (20°) using custom Python scripts.

#### Two-photon microscopy

Calcium signal was detected using a resonant two-photon microscope (Neurolabware) equipped with a Nikon 16× (NA = 0.8) water-emersion objective. Red (tdTomato) and green (GCaMP6s) fluorescence was stimulated using a Ti:Sapphire laser tuned to 900–920 nm (Mai Tai HP, Spectra-Physics). Data acquisition (10 Hz, 200–300 μm below meninges) was controlled by Scanbox software (Neurolabware). Visual stimuli were generated by custom Python software. The stimuli consisted of a blank (uniform luminance) condition, a full-field flicker (3 Hz) condition, and full-field drifting sinusoidal gratings (100% contrast) of 6–7 spatial frequencies (0.015–0.96, spaced logarithmically) and 12 directions (0°–330°, in 30° steps) at 3 Hz. Each condition consisted of a 1.5 s visual stimulus followed by a 1.5 s uniform gray screen. Each recording consisted of 10 trials and each trial consisted of randomized conditions. Stimuli were presented to one eye at a time using an occlude. The order of occlusion was chosen randomly for each session.

#### Immunofluorescence

Mice were transcardially perfused first with 1XPBS and then with fixative (4% PFA in 1xPBS). Brains were extracted, post-fixed, and cryopreserved in 30% sucrose. A freezing microtome was used to slice the brains into 50 µm thick sections. Fluorescent immunolabeling followed a standard indirect technique as described previously^62^. Briefly, free-floating sections were blocked in PBS supplemented with 0.2% Triton X100 (Sigma-Aldrich) and 5% goat serum (Sigma-Aldrich) and then incubated with primary overnight at 4 °C. Brain sections were stained with primary antibodies against: ionized calcium binding adaptor molecule 1 (IBA1) (1:1000; Catalog #019-19741, Wako). High resolution fluorescent images were obtained using a Leica TCS SPE-II confocal microscope and LAS-X software. For confocal imaging, three fields of view (FOV) per brain region was captured per mouse unless otherwise indicated. Total cell counts and spot analyses were obtained by imaging comparable sections of tissue from each animal at the 20X or 63X objective, at multiple z-planes, followed by automated analyses using Bitplane Imaris 7.5 spots.

### Data analysis

#### Intrinsic signal optical imaging analysis

Amplitude maps of cortical responses were extracted with Fourier analysis at the frequency of stimulus repetition^107^ using custom MATLAB (MathWorks) software. These maps were used to guide virus injection into bV1.

#### Two-photon analysis

Custom-written Matlab and Python code was used to remove motion artifact, place cell ROIs, extract calcium signal, and perform analyses as previously described^51^. Briefly, recordings were registered using an efficient algorithm that corrects for translational artifacts by minimizing the Euclidean distance between frames and a template image using a Fourier transform approach. ROIs were first placed manually for excitatory and inhibitory neurons. The somatic calcium signal at time t was determined as Fsoma(t) = Fsoma(t) − (R × Fneuropil(t))^88,93^. R was empirically determined to be 0.8 by comparing the intensity of GCaMP6s signal in blood vessels to the intensity of the neighboring neuropil. The neuropil signal Fneuropil(t) of each cell was measured by averaging the signal of all pixels outside of the cell and within an approximately 40 μm region from the cell center.

#### Responsiveness criteria

To determine a cell’s response to each condition, the ROI’s average calcium trace during a visual stimulus was normalized to the averaged calcium signal for the last 0.5 s of the preceding grey screen. The response to a given orientation θi was defined as the average response across 10 trials: F(θi). To assess neuronal tuning, we restricted our analysis to neurons that met the following two criteria. First, at each spatial frequency, responsiveness was determined using a one-way ANOVA (p < 0.01) across orientations against the blank condition. Second, the strongest evoked response had to be above the non-evoked calcium noise as determined using the blank stimulus: meanblank + (2xSDblank). A cell’s responsiveness was determined for each eye separately.

#### Peak spatial frequency

The peak spatial frequency was determined as the spatial frequency of the condition that elicited the strongest calcium response (Rmax). For statistical analysis, only the peak spatial frequency of the eye with the strongest evoked response was used. Population spatial frequency tuning curves were calculated first for each animal and then averaged across all mice in two ways. In the first method, spatial frequency tuning curves were centered around the peak spatial frequency and averaged. In the second method, spatial frequency tuning curves were averaged without shifting the curves.

#### Preferred orientation

The preferred orientation (θpref) was calculated along the peak spatial frequency, by calculating half the mean of the directional vectors weighted by the response F(θ) at each orientation as follows:

$${\theta }_{pref}=\frac{{\sum }_{i}{F\left({\theta }_{i}\right)e}^{{2}_{i}{\theta }_{i}}}{2{\sum }_{i}F\left({\theta }_{i}\right)}.$$

#### Orientation selectivity

Orientation selectivity was calculated at the peak spatial frequency using a method based on circular variance as follows:

$$OSI=\frac{\sqrt{{\sum }_{i}{F\left({\theta }_{i}\right)sin2{\theta }_{i})}^{2}+{\sum }_{i}{F\left({\theta }_{i}\right)cos2{\theta }_{i})}^{2}}}{{\sum }_{i}F({\theta }_{i})}.$$

Cells with OSI values below 0 and above 1 were excluded from analysis. For statistical analysis, only the orientation selectivity of the eye with the strongest evoked response was used. For each mouse, a population orientation tuning curve was calculated by centering tuning curves around the orientation that elicited the strongest response. Population tuning curves were averaged across all mice.

#### Ocular dominance index

Ocular dominance index was calculated as follows:

$$ODI= \frac{C-I}{C+I},$$

where C and I are the strongest contralateral and ipsilateral responses, respectively. An ODI of − 1 indicates a cell that only responds to stimulation through the ipsilateral eye. An ODI of 1 indicates a cell that only responds to stimulation through the contralateral eye. For neurons that were only responsive to monocular stimulation, the peak spatial frequency through the responsive eye was used to extract the strongest calcium signal through the non-responsive eye.

### Statistical analysis

GraphPad Prism (GraphPad Software v9) was used for Welsch’s t tests in Fig. 1 and a One-Way ANOVA controlling for the False Discovery Rate with a two-stage set-up method of Benjamini, Krieger, and Yekutieli test was used in Figs. 2 and 3. Custom Python routines were used for One-Way ANOVA testing for visual responsiveness and related selectivity calculations in Figs. 2 and 3. Data plotting were performed using Matlab scripts and GraphPad Prism.

## Data Availability

Data are available from the corresponding author on reasonable request.
