# Publisher Correction: Juvenile depletion of microglia reduces orientation but not high spatial frequency selectivity in mouse V1

**DOI:** 10.1038/s41598-022-27362-w

**Published:** 2023-01-24

**Authors:** Dario X. Figueroa Velez, Miguel Arreola, Carey Y. L. Huh, Kim Green, Sunil P. Gandhi

**Affiliations:** 1 Department of Pathology, Children’s Hospital Boston, Boston, MA 02115 USA; 2 Department of Neurobiology and Behavior, University of California, Irvine, CA 92697 USA; 3 Center for the Neurobiology of Learning and Memory, University of California, Irvine, Irvine, CA 92697 USA

Correction to: *Scientific reports* 10.1038/s41598-022-15503-0, published online 27 July 2022

The original version of this Article contained errors in Figure 3c and d, where the y-axis labels were incorrectly given. As a result,

Orientation Selectivity Index

now reads:

Spatial Frequency (cpd)

Orientation Selectivity Index

now reads:

Spatial Frequency (cpd)

The original Figure 3 and accompanying legend appear below.

The original Article has been corrected.

**Figure 3:**
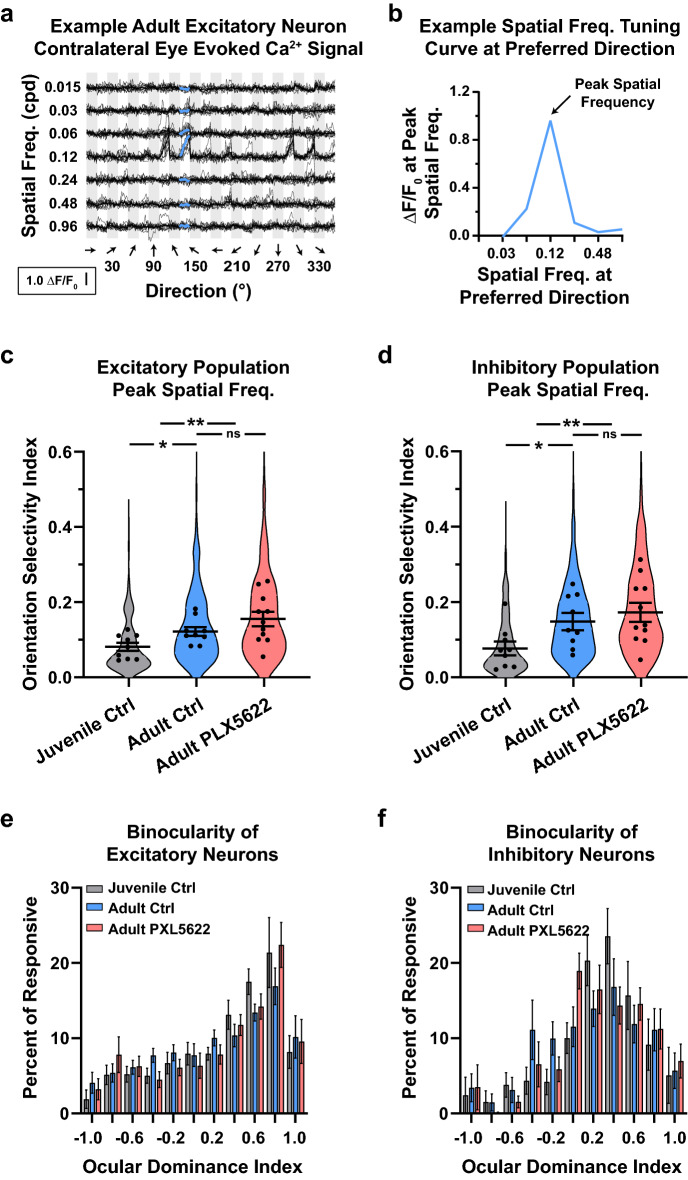
The caption to be typeset alongside it is: Microglia are not required for the developmental emergence of high spatial frequency tuning nor maintenance of normal binocularity in V1. (**a**) Example visually evoked calcium signal to presentations of stimuli through the contralateral eye in adult V1. The x-axis is organized by grating direction. The y-axis is organized by increasing grating spatial frequency. Thin and thick black lines represent individual and trial averaged traces, respectively. The blue line at 120° represents the averaged responses to different spatial frequencies directions at the neuron’s preferred direction. This trial-averaged trace was used to generate this neuron’s spatial frequency tuning curve and peak spatial frequency. (**b**) The spatial frequency tuning curve for the example neuron in (**a**). The peak spatial frequency for excitatory (**c**) and inhibitory (**d**) neurons. Violin plots represent the population distribution in juvenile (grey) and adult control (blue) mice, and adults on PLX5622 chow (red). Black circles represent an animal’s mean peak spatial frequency. (**c**) During normal development, excitatory neurons shift toward higher spatial frequencies (Juvenile Control = 0.08 ± 0.01 vs Adult Control = 0.12 ± 0.01, p = 0.035). The peak spatial frequency of mice fed PLX5622 (0.16 ± 0.02) was higher than juvenile (p = 0.002) and comparable to adult control mice (p = 0.253). (**d**) Like their excitatory counterpart, inhibitory neurons shift toward higher spatial frequencies during normal development (Juvenile Control = 0.08 ± 0.02 vs Adult Control = 0.15 ± 0.02, p = 0.043). The peak spatial frequency of mice fed PLX5622 (0.18 ± 0.03) was higher than juvenile (p = 0.006) and comparable to adult control mice (p = 0.450). Histogram of ocular dominance index for excitatory (**e**) and inhibitory (**f**) neurons in juveniles (grey), adults (blue), and mice lacking microglia (red). Microglia depletion did not alter the established binocularity of neurons in V1 (Juvenile Control = 0.45 ± 0.08 vs Adult Control = 0.30 ± 0.08 cpd, vs Adult PLX5622 = 0.36 ± 0.11). n_JuvenileControl_ = 9 mice, n_AdultControl_ = 9 mice, n_AdultPLX5622_ = 11 mice. Error bars represent the S.E.M.

